# Psychological distress in survivors of the 2010 Haiti earthquake

**DOI:** 10.1590/S1679-45082013000100004

**Published:** 2013

**Authors:** Melissa Simon Guimaro, Milton Steinman, Ana Merzel Kernkraut, Oscar Fernando Pavão dos Santos, Shirley Silva Lacerda

**Affiliations:** 1 Hospital Israelita Albert Einstein, São Paulo, SP, Brazil

**Keywords:** Depression, Anxiety, Disasters, Family relations, Stress, psychological

## Abstract

**Objective:**

To investigate the presence of depression and anxiety symptoms in survivors of the Haiti earthquake who were assisted by a healthcare team from the Hospital Israelita Albert Einstein, and to evaluate the impact that losing a family member during this catastrophe could have on the development of these symptoms.

**Methods:**

Forty survivors of the Haiti earthquake who were assisted by the healthcare team between February and March of 2010 were included in this study. All subjects underwent a semi-structured interview. The group was divided into Group A (individuals who had some death in the family due to the disaster) and Group B (those who did not lose any family member).

**Results:**

A total of 55% of the subjects had depression symptoms whereas 40% had anxiety symptoms. The individuals who lost a family member were five times more likely to develop anxiety and depression symptoms than those who did not.

**Conclusion:**

Catastrophe victims who lost at least one family member due to the disaster were more likely to develop anxiety and depression symptoms. To these individuals, as well as others showing psychological distress, should be offered early mental health care to help them cope with the great emotional distress inherent in these situations.

## RESUMO

**Objetivo:**

Investigar a presença de sintomas de depressão e ansiedade em sobreviventes do terremoto do Haiti, que foram atendidos pela equipe de saúde do Hospital Israelita Albert Einstein, e avaliar o impacto que a perda de um familiar durante a catástrofe pode causar no desenvolvimento desses sintomas.

**Métodos:**

Quarenta sobreviventes do terremoto do Haiti, atendidos pela equipe de saúde, entre fevereiro e março de 2010, foram incluídos neste estudo. Todos os indivíduos foram submetidos a uma entrevista semiestruturada. O grupo foi dividido em dois: Grupo A (que perderam um familiar na catástrofe) e Grupo B (aqueles que não tiveram perdas).

**Resultados:**

Um total de 55% dos indivíduos apresentavam sintomas de depressão e 40% de ansiedade. Os indivíduos que perderam familiares tinham cinco vezes mais probabilidade de desenvolver ansiedade e depressão do que aqueles não tiveram perdas.

**Conclusão:**

As vítimas de catástrofes que perderam pelo menos um familiar no desastre têm maior probabilidade de desenvolver sintomas de depressão e ansiedade. A esses indivíduos, assim como outros que demonstravam estresse psicológico, devem ser oferecidos, precocemente, cuidados de saúde mental, para ajudá-los a suportar o grande estresse emocional inerente a essas situações.

## INTRODUCTION

The impact of the natural catastrophes that occurred recently has been greater than ever before. With an increasing population residing in risky areas, the consequences of such catastrophes have aggravated. Current studies show that these disasters lead to highrisk situations for the development of functional and psychological problems^(1,2)^. Soon after enduring a traumatic event, many people develop symptoms of psychological stress. If the trauma affects the routine of the victim, this individual may systematically avoid any contact with the incident, making the symptoms chronic^(3)^.

Natural disasters are one of the main priorities of the mental health community, since the survivors may need psychological support for at least 1 year after the event^(4,5)^ . Many studies showed a high prevalence of psychiatric disorders in survivors of natural disasters, indicating the great negative impact of the emotional trauma caused by these situations^(6–10)^ .

On January 12, 2010, an earthquake measuring 7.0 on the Richter scale struck Haiti. The epicenter was about 16 miles west of its capital, Port-au-Prince. The earthquake caused widespread loss of life and damage, and many organizations responded to appeals from the Haitian people for humanitarian aid. This work was done at Fond Parisien Disaster Recovery Center from January to May 2010, in Fond Parisien region, located about 37km away from Port-au-Prince^(11)^ . The center was coordinated by the Harvard Humanitarian Initiative and it was created to accommodate thousands of injured earthquake victims who underwent surgery at a nearby hospital. This displacement camp provided a temporary home for over 2,000 injured survivors and their families. Approximately 725 people from over 16 countries volunteered their time at the center, where over 350 limb-saving operations were conducted^(11)^ .

By the time the present study ended, there were over 250 studies available in PubMed on the earthquake that occurred in Haiti in 2010, however, none investigated the psychological aspects of the survivors of this recent catastrophe.

## OBJECTIVE

To investigate the occurrence of depression and anxiety in a group of survivors of the Haiti earthquake who were seen by a health-care team from Hospital Israelita Albert Einstein (HIAE), as well as to evaluate the impact that losing a family member during this catastrophe could have on the development of depression and anxiety symptoms.

## METHODS

Forty survivors of the Haiti earthquake who were assisted by the HIAE healthcare team, from Brazil, between February and March of 2010 were included in this study. All subjects underwent a semi-structured interview for collection of demographic data, medical history, recollections of the event and signs of emotional distress. A clinical interview, based on the Hamilton Depression Scale^(12)^ and Beck Anxiety Inventory^(13)^, was done to investigate the presence or absence of depression and anxiety symptoms. After data collection and correlation analysis, the group was divided into two. Group A comprised individuals who had some death in the family and Group B of those who did not lose any family member due to the disaster.

We used the Statistical Package for the Social Sciences (SPSS) for Windows (16.0) for statistical analysis, as well as the Spearman correlation, partial correlation, Fisher's exact, and χ^2^ tests.

All subjects provided informed consent to participate in the study. Individuals showing emotional distress were referred to further orientation and/or supportive interventions and, when needed, they were referred to psychiatric consultation-liaison.

## RESULTS

Subjects were on average 34.70 (standard deviation, SD=19.88) years old. Eighty percent (n=32) were female, 60% (n=24) were single, 75% (n=30) protestant, and 55% (n=22) had completed middle school. The average time for receiving first medical aid after the earthquake was 4.33 (SD=4.20) days. The most common diagnoses were fractures (n=23; 57.5%), soft tissue lesions (n=9; 22.5%), and amputations (n=8; 20%). No significant difference was found regarding the presence or absence of symptoms of depression and anxiety in these three diagnostic groups. As for recollections of the event, 72% (n=29) reported that they saw dead bodies and 52% (n=21) recollected the loss of a family member.

After the clinical interview, 22 (55%) subjects presented depression symptoms and 16 (40%) presented anxiety symptoms. Correlation analysis revealed that the death of a family member was significantly associated with both anxiety (rho=0.368; p=0.020) and depression symptoms (rho=0.374; p=0.028). Seeing dead bodies did not correlate with the presence of these symptoms (anxiety: rho=0.046; p=0.779; depression: rho=0.231; p=0.152).

Groups A and B did not show significant differences in regard to the age of the individuals (Group A: M=35.71, SD=20.69 years old; and Group B M=33.57, SD=19.44; p=0.739) or education level (p=0.356). However, Group B had significantly more males than Group A (p=0.017). Nevertheless, no significant differences in anxiety or depression symptoms correlated with sex and amputation and seeing dead bodies.

Table 1 shows that the differences for depression and anxiety symptoms were significant between groups. Note that those who lost a family member were five times more likely to develop anxiety and depression symptoms than individuals in Group B.

**Table 1:** Presence of anxiety and depression symptoms between groups

		Death		χ^2^	Odds ratio	95%CI
		Yes	No			
Anxiety symptoms	Yes	12	4	5.41*	5.00	1.23-20.30
	No	9	15			
Depression symptoms	Yes	15	7	4.82*	4.28	1.13-16.18
	No	6	12			

* p<0.05.

95%CI: 95% confidence interval.

## DISCUSSION

Current studies indicate that a great number of individuals will develop emotional distress, such as anxiety and depression symptoms, and difficulties in situations of disaster^(1,2,4,6–10)^ . Although highly disturbing, the emotional reaction to these events is seen as a human response to great adversity^(14)^ . In this study, the individuals who lost at least one family member presented higher chances of developing anxiety and depression symptoms, which seem to be related to the mourning process. Furthermore, no risk was found for depression and anxiety symptoms in any of three medical diagnoses. We think that associations between symptoms and diagnoses were not found because, at the moment of analysis, patients had not returned to their routine and they had not had enough time to feel the impact of limitations in their lives.

According to the current literature on this subject, females had greater psychological distress^(7,15)^ . However, in our study, we found anxiety and depression symptoms correlated with death in the family, regardless of the gender of the subject evaluated.

The high prevalence of anxiety and depression symptoms found in the Haitians here was similar to those found in other studies done with survivors of natural catastrophes^(15–18)^ . In our study, the fact that these individuals were evaluated 1 month after the event suggested that they may have developed acute stress disorder^(10)^ . Therefore, the lack of proper treatment may lead to the development of post-traumatic stress disorder in this population. Patients who had recollections of the disaster presented more symptoms than those who did not. Thus, recurring memories may be associated with avoidance behaviors, which seem to favor the development of depression symptoms^(19,20)^ .

The experiences the survivors endured at the time of the disaster as well as the availability of early and effective interventions should be considered to increase the resilience capacity of individuals and also their emotional strength to these situations to the best of their ability^(6,10)^ . It should be noticed that besides physical care most individuals in these situations also need mental health care. The affected community requires psychosocial support not only to reduce psychological distress but also to facilitate physical rehabilitation^(8,9)^ .

### Study limitations

Despite the fact that few studies investigated the psychological aspects of survivors from this recent catastrophe in Haiti, and the great relevance of this subject, there are some limitations in this study that must be considered. It had a small and not randomized sample, and did not represent the entire population. Because of the lack of organization, extreme poverty and suffering in the country in the aftermath, the work environment at the camp to support survivors and the way data was collected did not provided an appropriated control to select subjects. Nevertheless, the clinical interview based on standard scales showed sensible and trustable to indicate the presence or absence of depression and anxiety symptoms.

## CONCLUSION

Catastrophe victims who lost at least one family member due to the disaster have greater chance of developing anxiety and depression symptoms. To these individuals, as well as to others showing psychological distress, mental health care should be offered early enough to help them cope with the great emotional distress inherent in these situations.
